# Blocking Serum Amyloid-P Component from Binding to Macrophages and Augmenting Fungal Functional Amyloid Increases Macrophage Phagocytosis of *Candida albicans*

**DOI:** 10.3390/pathogens11091000

**Published:** 2022-09-01

**Authors:** Stephen A. Klotz, Nicole Bradley, Peter N. Lipke

**Affiliations:** 1 Division of Infectious Diseases, University of Arizona, Tucson, AZ 85724, USA; 2 Department of Anatomy and Physiology, San Jacinto College, Houston, TX 77049, USA; 3 Department of Biology, Brooklyn College of the City University of New York, New York, NY 11210, USA

**Keywords:** macrophage skewing, pathogen recognition, innate immunity

## Abstract

*Candida*-macrophage interactions are important immune defense responses associated with disseminated and deep-seated candidiasis in humans. Cells of *Candida* spp. express functional amyloids on their surfaces during the pathogenesis of disseminated candidiasis. These amyloids become decorated with serum amyloid P-component (SAP) that binds to *Candida* cells and macrophages and downregulates the cellular and cytokine response to the fungi. In this report, further characterization of the interactions of SAP and fungal functional amyloid are demonstrated. Blocking the binding of SAP to macrophage FcγR1 receptors increases phagocytosis of yeast cells; seeding a pro-amyloid-forming peptide on the yeast cell surface also increases phagocytosis of yeasts by macrophages; and, lastly, miridesap, a small palindromic molecule, prevents binding of SAP to yeasts and removes SAP that is bound to *C. albicans* thus, potentially increasing phagocytosis of yeasts by macrophages. Some, or all, of these interventions may be useful in boosting the host immune response to disseminated candidiasis.

## 1. Introduction

The serendipitous finding in human postmortem tissue specimens of serum amyloid-P component (SAP) bound to the cell surface of *Candida* provided evidence that *Candida* functional amyloids were expressed during the pathogenesis of candidiasis and that these amyloids were recognized by human SAP [1]. It has been proposed that functional amyloids are the rule rather than the exception in cellular biology [2] and are ubiquitous in nature [3]. SAP is a phylogenetically conserved serum protein that binds avidly to extracellular and intracellular amyloids and in doing so renders the “misfolded” pathological protein immunologically inert [4]. We have found that SAP bound to *C. albicans* influences many macrophage functions, e.g., inhibiting phagocytosis of yeasts, inhibiting macrophage secretion of interferon-γ, TNF-α, IL-6, and, contrarily, increasing IL-10 secretion. These effects skew the macrophages towards the more quiescent M2 state [5]. It turns out that SAP binding to fungal functional amyloids is a feature of many deep-seated fungal infections [6]. However, much remains to be determined regarding whether SAP is protecting the host or alternatively, by binding to the microbe, protecting the pathogen [6,7,8]. 

SAP circulates in the blood as a pentamer. In human females it is found at a constant level of ~15 µg/mL, and in males at ~30 µg/mL [9]. SAP constitutes roughly 30% of the mass of amyloid deposits and is not degraded by proteases. Thus, it protects pathological amyloid aggregates that grow over time [10]. Therefore, removing SAP from the serum and tissue has been one approach to the treatment of the amyloidoses [4,9]. It is in this connection with amyloidosis that miridesap was developed. It is a palindrome of two proline residues (CPHPC) that dimerizes SAP to a decamer and facilitates its clearance from serum in vivo [11]. In combination with anti-SAP antibodies, miridesap has successfully cleared human amyloid deposits [12]. 

We have tested the effects of blocking SAP from binding to macrophages using compounds that block SAP binding to FcγR1 receptors on the macrophages; also, we tested peptides that alter the amount of fungal amyloid on the yeast cell surface and the effect on phagocytosis; finally, we have tested the effect of miridesap on SAP binding to yeast cells. The results from these experiments were consistent with the idea that SAP, when bound to fungal surface amyloids, protects the pathogen from the host. 

## 2. Methods

### 2.1. Macrophages

Human monocytes were isolated from whole blood using the SepMate-50 system (Stemcell Technologies, Seattle, WA, USA). The protocol was approved by the University of Arizona IRB. Cells were then cryopreserved. Peripheral blood mononuclear cells (PBMCs) from normal (healthy) adult controls were placed in complete RPMI (RPMI, 10% fetal bovine serum, penicillin/streptomycin, and DNase (3 ng/mL)) and thawed to 37 °C. Cells were collected by centrifugation, resuspended in X-VIVO-15 (Lonza, Walkersville, MD, USA), a serum-free medium, plated into 96-well plates (Fisher Scientific) (10^5^ cells in 100 μL medium/well), and placed in an incubator overnight at 37 °C with 5% CO2. On the following day PBMCs were washed with serum-free medium and resuspended in fresh medium, human Macrophage Colony Stimulating Factor (Sigma-Aldrich, St. Louis, MO, USA) was added to each well (1 ng/mL), and cells incubated for 5 days at 37 °C with 5% CO_2_ [5]. Rat bone marrow macrophages (ScienCell Research Laboratories, Carlsbad, CA, USA) were treated in the same manner with the exception that 10% rat serum was provided in the RPMI as per manufacturer’s directions.

### 2.2. Fungi

A clinical isolate of *Candida albicans* (Banner University Medical Center, Tucson, AZ, USA) and *C. albicans* SC5314 were used in these experiments. The strains were maintained on YPD agar (RPI, Mount Pleasant, IL, USA). For experiments, a loopful of fungi was added to 5 mL YPD broth (Life Technologies Corp., Carlsbad, CA, USA) and incubated overnight at 26 °C with shaking. After 24 h, the yeasts were collected by centrifugation at 1200× *g* for 5 min, resuspended, and washed thoroughly in Tris-buffered saline with 2 mM Ca^2+^ (TBS-C). This was repeated three times, and cells resuspended to 10^9^ cells/mL in TBS-C. 

### 2.3. Macrophage–Yeast Interactions

Wells containing 5-day-old macrophages in 96-well plates were washed with serum-free medium, and 100 μL of fresh serum-free medium and 1 μL containing 10^6^ previously washed yeasts suspended in TBS-C were added to each well with or without additives as explained. For other experiments, alongside the addition of *C. albicans* alone to wells, *C. albicans* was also incubated in 100% normal human male AB serum (Innovative Research, Novi, MI, USA) or SAP (Millipore, Temecula, CA, USA) at 30 μg/mL or rat serum for 1 h at 4 °C, washed with TBS-C, resuspended in serum-free medium, warmed to 37 °C and added to wells or chambers. The cocultures were incubated at 37 °C on a rocking shaker for 30 min. The yeast/macrophage ratio was 100:1. A 30 min incubation of yeasts with macrophages was employed to minimize the number of yeasts forming germ tubes. Following incubation of yeasts with macrophages, the wells were washed gently three times with TBS-C, and the wells or slides stained with Wright-Giemsa. Stained wells and slides were observed by light microscopy, and a phagocytic index (number of yeasts phagocytosed/50 macrophages/well) determined for each replicate in 96-well plate. Each assay was repeated a minimum of three times. Methods of Du and Calderone were adapted to our specific uses [13].

### 2.4. SAP and Other Additives

Human SAP or buffer-exchanged SAP in serum-free medium was added to wells to a final concentration of 30 μg/mL. Other additives included miridesap at stated concentrations.

### 2.5. The SAP-Competitor Compounds

Compounds were stored in 100% DMSO and were diluted in RPMI to 100 µM (gift of RH Gomer). Where indicated, SAP was added at day 7 to the rat macrophages to 5 µg/mL. Inhibitory compounds were added at the same time to 1 µM final concentration, respectively [14]. 

### 2.6. Pro-Amyloid and Anti-Amyloid Peptides

The pro-amyloid peptide, SNGIVIVATTRTV, and the anti-amyloid peptide, SNGI**N**IVATTRTV [15,16] were added to the yeasts or macrophages as 20 mg/mL final concentration for 1 h. Yeast cells were then washed, and the phagocytosis assay conducted.

### 2.7. Flow Cytometry

Fungi were cultured overnight (*C. albicans*) as described above, collected by centrifugation, and resuspended in TBS-C. Cells were incubated in undiluted normal human male AB serum, rat serum or in TBS-C with 30 μg/mL SAP for 1 h at 4 °C, washed three times by centrifugation, and resuspended in TBS-C. Cells were then incubated in TBS-C with 1:100 rabbit polyclonal anti-human SAP antibodies (PA5-24171; Invitrogen), or anti-rat SAP antiserum. Yeasts were then washed three times and incubated with 1:200 fluorescein-labeled goat anti-rabbit or anti-rat antibody (Invitrogen) for 30 min, washed three times, and resuspended in TBS [5]. Cell fluorescence was read on a BD LSR II flow cytometer (BD Biosciences, San Jose, CA, USA) and analyzed using Flowjo version 10 (Ashland, OR, USA). 

### 2.8. Statistics

All population totals were graphed using median values and 95% confidence intervals or means with standard errors. An unpaired, nonparametric Mann–Whitney test or a basic unpaired *t* test was used to determine statistical significance between different populations and treatment conditions. The software used was Prism version 7 (Graphpad, San Diego, CA, USA). 

## 3. Results

### 3.1. Compounds That Inhibit SAP Binding to Macrophages Enhance Phagocytosis

**Figure 1:**
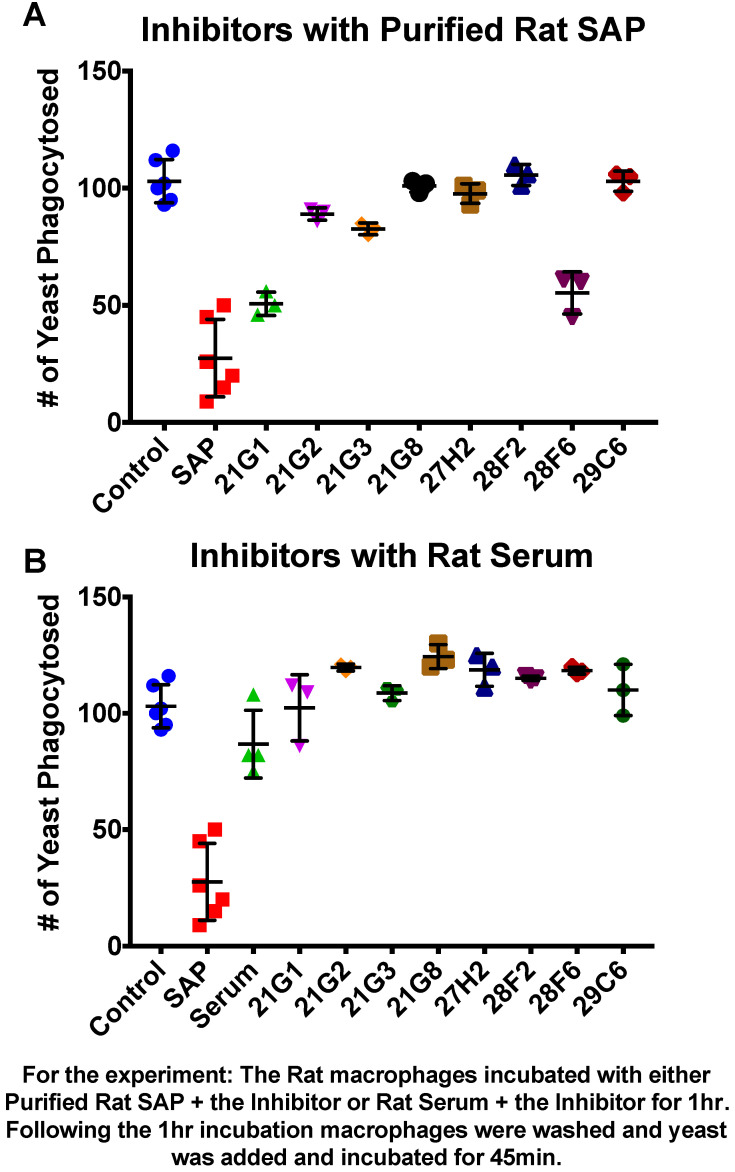
Inhibitors of serum amyloid P component binding to rat macrophages increases *Candida albicans* yeast phagocytosis by rat macrophages. SAP was provided as pure rat SAP (**A**) or from rat serum, (**B**) (means and standard deviations of 3 independent trials are shown). See reference [14] for more information about the inhibitors.

The addition of SAP to macrophages diminishes the number of yeast that macrophages phagocytose about 10-fold and skews macrophages towards the M2-tolerant state [5]. Therefore, SAP binding to macrophages leads to a less acute inflammatory response to pathogens. Xiang et al. [14] screened a library for compounds that inhibit SAP binding to the macrophage FcγRI receptor and discovered several compounds that were effective at mM concentrations. We tested the eight most effective inhibitors from their study [14] to determine whether inhibition of SAP binding to FcγR1 would increase yeast phagocytosis. Rat macrophages and rat SAP were used. Rat SAP has the advantage that it is more like human SAP than mouse, being constitutively secreted into serum like humans, whereas mouse SAP is an acute phase reactant. Figure 1 shows that in the absence of SAP, rat macrophages phagocytosed over 100 yeasts/50 macrophages, whereas in the presence of rat SAP, ~25 yeasts/50 cells were phagocytosed, a pattern like that of human SAP/macrophage interactions [5]. Each of the inhibitors (1 µM) was added to macrophages treated with either purified rat SAP or rat serum. The macrophages were then washed and tested for phagocytosis of *C. albicans* yeast cells. The tested compounds restored macrophage phagocytosis to levels approaching control levels (~100 yeasts/50 cells) by preventing SAP binding to the macrophage. All eight compounds were fully active against SAP in serum, whereas two compounds showed less activity against macrophages treated with purified SAP. 

### 3.2. ‘Seeding’ Amyloid on the Yeast Surface Enhances Phagocytosis of Yeasts

**Figure 2:**
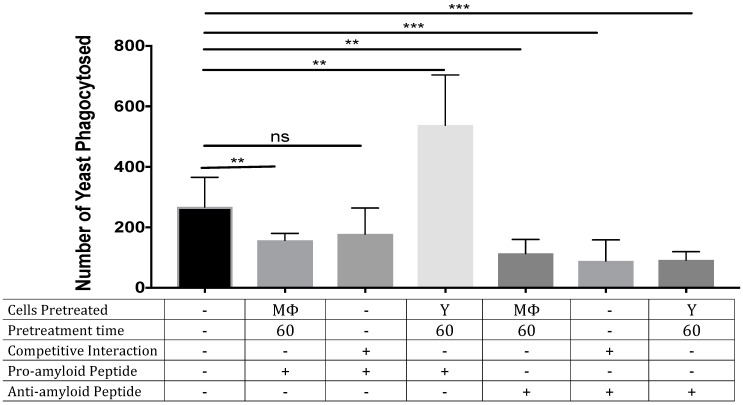
Increasing *Candida albicans* yeast phagocytosis by human macrophages by treatment with pro-amyloid peptide. Yeasts (Y) or macrophages (M) were either pre-treated with peptides or peptides were added to the yeasts and macrophages at the same time (competitive experiments) for the specified time periods (means and standard deviations for 6 independent trials are shown). **: *p* < 0.01; ***: *p* < 0.001.

Fungi express functional amyloids in the adhesins on their cell surface [1,5,15,16], and these amyloids potentiate phagocytosis. Specifically, the presence of the amyloid sequence ^324^GIVIVA^329^ within *C. albicans* adhesin Als5 induces high levels of phagocytosis of *S. cerevisiae*, whereas Als5 with the non-amyloid sequence ^324^GI**N**IVA^329^ does not. Exogenous peptides of similar sequence bind to the amyloid sequence to either increase or decrease the amount of surface amyloid [16,17]. Nevertheless, on the surface of *C. albicans* there are many adhesins with amyloidogenic sequences, and we do not know whether the specific ^324^GIVIVA^329^ amyloid sequence (expressed in adhesins Als1, Als3, and Als5) affects macrophage response on the complex surface of *C. albicans*. Therefore, we tested whether potentiation or inhibition of amyloid formation would have similar effects in *C. albicans*. Pre-treating *C albicans* with exogenous pro-amyloid peptide SNGIVIVATTRTV increased macrophage phagocytosis by 89% (Figure 2). In contrast, the anti-amyloid peptide SNGINIVATTRTV decreased phagocytosis by about 61%. Adding either peptide during the phagocytosis assay inhibited phagocytosis, as did pre-treatment of the macrophages with either peptide. Therefore, peptide enhancement of yeast surface amyloid promoted phagocytosis, and peptide inhibition of yeast surface amyloid was inhibitory. Interestingly, both peptides inhibited phagocytosis when macrophages were exposed to them in solution. 

### 3.3. Miridesap Successfully Removes SAP from the Yeast Cell Surface

**Figure 3:**
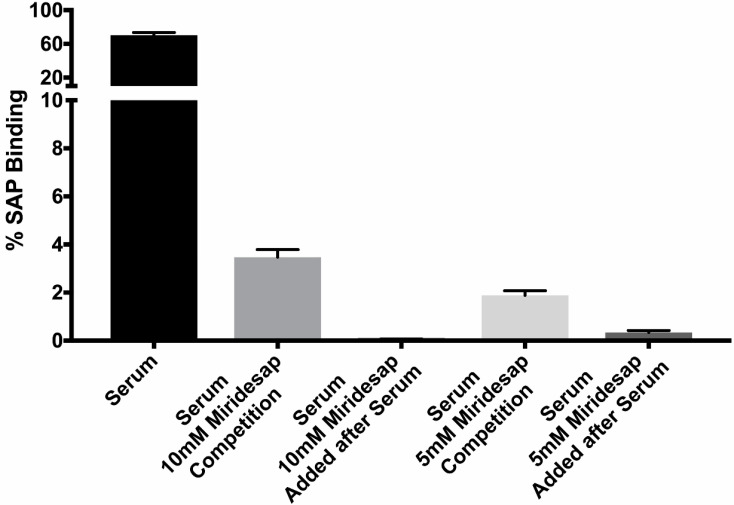
Effect of miridesap on SAP binding to *C. albicans.* Yeasts were added to human serum or human serum plus miridesap (at 5 or 10 mM) for 1 h; washed, and the amount of rat SAP bound to the yeasts determined by flow cytometry. (Means and standard deviation shown for 3 independent trials.

SAP binding decreases macrophage phagocytosis of *C. albicans* yeast more than 10-fold [5]. Therefore, we investigated whether miridesap could prevent binding of SAP to yeasts and whether the drug could remove SAP from yeasts once SAP is bound. Yeasts were incubated in 10% human serum for 1 h at 4 °C, then washed and assayed for SAP binding to the yeast cells by flow cytometry. SAP bound to ~75% of the yeasts (as measured by flow cytometry). Adding miridesap (5 mM or 10 mM) with the serum (labeled as “Competition”) or after binding of SAP to the yeasts reduced the number of yeast cells positive for SAP to 0–3% (Figure 3). The same results were obtained using rat SAP and miridesap, the drug preventing SAP from binding to yeasts in the competitive assay and removing previously bound rat SAP from the yeast cell surface (data not shown).

## 4. Discussion

There are complex interactions between macrophages, SAP, and the fungal human pathogen *C. albicans.* Our study of the effects of exogenous effectors of the SAP-macrophage-fungal interaction reveals some of these specific effects, and points to possible therapeutic interventions. Specifically, SAP binding to the fungi decreases phagocytosis, but the effects of SAP on macrophages are more complex. 

Phagocytosis of *Candida albicans* by human macrophages is an important component of the host response to disseminated candidiasis. In the pathogenesis of disseminated candidiasis, fungi gain access to all tissues through the vascular system where the macrophages encounter the fungi [18]. Host macrophages are tasked with orchestrating an immunological defense by secreting cytokines as well as destroying fungi in phagocytic vacuoles. Because *Candida* spp. possess cell surface functional amyloid to which SAP binds, it is possible that the presence of SAP may affect the host response to the pathogen [5]. 

Nouresadeghi et al. showed that binding of human SAP to bacteria shortened the lifespan of mice given fatal doses of bacteria intravenously [7]. However, in that short-lived model, the polymorphonuclear leukocytes were likely the most important host defense cells. Studies of murine alveolar macrophages treated with SAP demonstrated reduced phagocytosis of *Mycobacterium tuberculosis* [19], and a similar outcome was found with human monocyte/macrophages and other mycobacteria [14]. These results parallel our findings. Nevertheless, SAP is not always deleterious, e.g., it ameliorates and stops the progression of interstitial pulmonary fibrosis in humans [20] and dampens the cytokine storm in mice caused by inhalation of SARS-CoV2 mRNA [21]. Amelioration of disease is accomplished by SAP binding to the macrophage and skewing macrophage populations toward the M2 phenotype [21,22]. 

The binding of SAP to *C. albicans* cells is dependent on SAP recognition of amyloid-like cross-β bonded structures on the yeast cell surface [4,5,6]. These adhesins are clustered on the cell surface to form high-avidity adhesive patches that mediate binding to the host. [15,16,17]. Amyloid-like cross-β bonds also bind *C. albicans* cells together to make robust cell-cell bonds in vitro and in the host [16]. Miridesap is a potent inhibitor of SAP binding to the fungus and can even dissociate pre-bound SAP (Figure 3). The consequences of this stripping would be greatly increased phagocytosis of the fungi since amyloid-like structures on the yeast are rapidly recognized by macrophages and trigger subsequent phagocytosis. This finding was reinforced when the fungi were treated with the peptide SNGIVIVATTRTV. This peptide binds to and enhances surface amyloid formation [15,17], leading to the increased phagocytosis shown in Figure 2. In contrast, treatment of the yeast with the anti-amyloid peptide SNGINIVATTRTV decreased surface amyloid structures and consequently inhibited phagocytosis more than 3-fold (Figure 2). Therefore, fungal surface amyloids are recognized by macrophages [5,6] and act as a pro-phagocytosis signal. However, in the presence of SAP, the SAP binds to these same surface amyloid-like structures, masking the yeast and inhibiting phagocytosis [5] (Figure 2).

What role SAP plays in disseminated candidiasis is unknown, in spite of the fact that SAP avidly binds to fungi in human disease and contributes to the biofilm. [1,6] Miridesap is a palindromic molecule composed of two prolines and developed as a drug for use in the therapy of amyloidosis [9]. In serum it binds two molecules of SAP forming a decamer that is removed from the circulation by the liver [11]. SAP is in equilibrium between the serum and tissue and with its removal from serum leads to removal of SAP from amyloidotic deposits. It is shown here that the drug is capable of preventing SAP from binding to yeasts as well as stripping yeasts of bound SAP (Figure 3). Knowing that miridesap was capable of removing SAP bound to amyloid deposits, Pepys proposed using the drug as possible adjunctive therapy in deep-seated fungal infections [23]. We are currently conducting a mouse model of disseminated candidiasis incorporating treatment with miridesap.

The binding of SAP to macrophages promotes polarization to a tolerant, non-inflammatory M2 state, [5,14,22] that phagocytoses fewer yeast (Figure 1). Therefore, it is not surprising that inhibitors of SAP binding to the macrophage FCγRI receptor reverse this polarization [14] and promote phagocytosis (Figure 1). Because phagocytosing macrophages polarize to the pro-inflammatory M1 state, SAP has two reinforcing effects on macrophages [5]. First, SAP itself reduces macrophage polarization to the pro-inflammatory M1 state. Second, SAP bound to *C. albicans* masks the surface amyloids and therefore it also reduces M1 polarizing phagocytosis. Therefore, the presence of SAP inhibits M1 polarization and promotes polarization to the more tolerant M2 state. Thus, inhibitors of SAP binding to either yeast or macrophages increase phagocytosis, and the consequent polarization to the pro-inflammatory M1 state. We are currently assessing whether inhibition of SAP binding affects pathogenesis in the mouse systemic candidiasis model. 

## Data Availability

Data stored in University of Arizona and San Jacinto College.
